# The possible link between the elevated serum levels of neurokinin A and anti-ribosomal P protein antibodies in children with autism

**DOI:** 10.1186/1742-2094-8-180

**Published:** 2011-12-21

**Authors:** Gehan A Mostafa, Laila Y AL-Ayadhi

**Affiliations:** 1 Autism Research and Treatment Center, AL-Amodi Autism Research Chair, Department of Physiology, Faculty of Medicine, King Saud University, Riyadh, Saudi Arabia; 2 Department of Pediatrics, Faculty of Medicine, Ain Shams University, Cairo, Egypt

**Keywords:** Anti-ribosomal P protein antibodies; autism, autoimmunity, neurokinin A

## Abstract

**Background:**

Neurogenic inflammation is orchestrated by a large number of neuropeptides. Tachykinins (substance P, neurokinin A and neurokinin B) are pro-inflammatory neuropeptides that may play an important role in some autoimmune neuroinflammatory diseases. Autoimmunity may have a role in the pathogenesis of autism in some patients. We are the first to measure serum neurokinin A levels in autistic children. The relationship between serum levels of neurokinin A and anti-ribosomal P protein antibodies was also studied.

**Methods:**

Serum neurokinin A and anti-ribosomal P protein antibodies were measured in 70 autistic children in comparison to 48 healthy-matched children.

**Results:**

Autistic children had significantly higher serum neurokinin A levels than healthy controls (P < 0.001). Children with severe autism had significantly higher serum neurokinin A levels than patients with mild to moderate autism (P < 0.001). Increased serum levels of neurokinin A and anti-ribosomal P protein antibodies were found in 57.1% and 44.3%, respectively of autistic children. There was significant positive correlations between serum levels of neurokinin A and anti-ribosomal P protein antibodies (P = 0.004).

**Conclusions:**

Serum neurokinin A levels were elevated in some autistic children and they were significantly correlated to the severity of autism and to serum levels of anti-ribosomal P protein antibodies. However, this is an initial report that warrants further research to determine the pathogenic role of neurokinin A and its possible link to autoimmunity in autism. The therapeutic role of tachykinin receptor antagonists, a potential new class of anti-inflammatory medications, should also be studied in autism.

## Background

Neurogenic inflammation encompasses a series of vascular and non-vascular inflammatory responses, triggered by the activation of primary sensory neurons, with a subsequent release of inflammatory neuromediators. This results in a neurally mediated immune inflammation [1,2]. Neuromediators are mainly released from neurons. Immune and/or structural cells are secondary sources of these mediators during immune inflammation [3,4]. Neuromediators include neurotrophins and neuropeptides [4].

Neurogenic inflammation is orchestrated by a large number of neuropeptides mainly including tachykinins. Tachykinins (substance P, neurokinin A and neurokinin B) have been considered as a group of neuropeptides which are released from the excitatory part of the nonadrenergic, noncholinergic excitatory nervous system nerves after exposure to allergens. The biological activity of tachykinins depends on their interaction with three specific tachykinin receptors, neurokinin (NK)1 (specific for substance P), NK2 (specific for neurokinin A) and NK3 (specific for neurokinin B) receptors [5-7]. Tachykinin receptor antagonists are a potential new class of anti-inflammatory medicaions in immune-mediated diseases [8-10].

Autoimmunity may have a role in the pathogenesis of autism in a subgroup of patients. This may be indicated by the presence of brain-specific auto-antibodies in some autistic children [11-17]. There is also an increase in the frequency of autoimmune disorders among autistic families [18-23]. Inspite of the fact that the origins of autoimmunity in autism are unknown, the major histocompatibility complex genes and their products might be involved [21,24,25].

Anti-ribosomal P protein antibodies are one group of potentially pathogenic autoantibodies that has a specificity for the functional center of the ribosomal P proteins which is a family of highly conserved acidic phosphoproteins primarily located on the stalk of the large (60 s) ribosomal subunit [26]. They bind 3 ribosomal proteins identified as P0, P1 and P2 (38, 19 and 17-kDa, respectively) by recognizing a certain epitope found in those 3 proteins. Several possible pathogenic mechanisms for these antibodies in some autoimmune diseases include their binding to epitopes on the cell membrane surface, intracellular penetration, inhibition of protein synthesis, production of pro-inflammatory cytokines and cell apoptosis [27].

Evidence for an interaction between chronic inflammation in autoimmune diseases and neural dysfunction points to an involvement linking the nervous and the immune system. In this context, neuropeptides, including tackykinins and neurotrophins have been recognized as key mediators of neuro-immune interactions in some autoimmune diseases [28]. Thus, investigations regarding the development of pharmacological compounds specifically targeting these molecules could be of interest [29].

This study was the first to measure serum neurokinin A levels in a group of autistic children. The relationship between serum levels of neurokinin A and anti-ribosomal P protein antibodies was also studied.

## Methods

### Study population

This cross-sectional study was conducted on 70 children who had autism. They were recruited from the Autism Research and Treatment Center, Faculty of Medicine, King Saud University, Riyadh, Saudi Arabia. Patients were fulfilling the criteria of the diagnosis of autism according to the 4^th ^edition of the Diagnostic and Statistical Manual of Mental Disorders [30]. The autistic group comprised 55 males and 15 females. Their ages ranged between 4 and 12 years (mean ± SD = 8.10 ± 2.52 years).

Exclusions criteria:

1- Patients who had associated neurological diseases (such as cerebral palsy and tuberous sclerosis) and metabolic disorders (eg. Phenylketonuria) were excluded form the study.

2- Patients with associated allergic, inflammatory or autoimmune disorders.

3- Patients who were receiving any medications.

The control group comprised 48 age- and sex- matched apparently healthy children (37 males and 11 females). They were the healthy older siblings of the healthy infants who attend the Well Baby Clinic, King Khalid University Hospital, Faculty of Medicine, King Saud University, Riyadh, Saudi Arabia for routine following up of their growth parameters. The control children were not related to the children with autism, and demonstrated no clinical findings suggestive of immunological or neuropsychiatric disorders. Their ages ranged between 6 and 11 years (mean ± SD = 8.79 ± 2.89 years).

The local Ethical Committee of the Faculty of Medicine, King Saud University, Riyadh, Saudi Arabia, approved this study. In addition, an informed written consent of participation in the study was signed by the parents or the legal guardians of the studied subjects.

### Study measurements

#### Clinical evaluation of autistic patients

This was based on clinical history taking from caregivers, clinical examination and neuropsychiatric assessment. In addition, the degree of the disease severity was assessed by using the Childhood Autism Rating Scale (CARS) [31] which rates the child on a scale from one to four in each of fifteen areas (relating to people; emotional response; imitation; body use; object use; listening response; fear or nervousness; verbal communication; non-verbal communication; activity level; level and consistency of intellectual response; adaptation to change; visual response; taste, smell and touch response and general impressions). According to the scale, children who have scored 30-36 have mild to moderate autism (n = 34), while those with scores ranging between 37 and 60 points have a severe degree of autism (n = 36).

#### Serum assessment of neurokinin A

Serum levels of neurokinin A were evaluated with an enzyme-linked immunosorbent assay (ELISA) kit which is highly sensitivie to neurokinin A. Neurokinin A like immunoreactivity was measured using an antibody that has originally been isolated from porcine spinal cord. It shows 100% cross reactivity to neurokinin A with little reactivity to other tachykinins (Peninsula laboratories, 611 Talorwat, Belmont, CA, USA). To increase accuracy, all samples were analysed twice in two independent experiments to assess the interassay variations and to ensure reproducibility of the observed results (P > 0.05). No significant cross-reactivity or interference was observed.

#### Measurement of serum anti-ribosomal P protein antibodies

Serum total IgG and IgM anti-ribosomal P protein antibodies were measured by ELISA using ribosomal P peptide-bovine serum albumin conjugate as an antigen (Nunc immuno module F8 maxisorp; Nunc. Roskilde, Denmark). To increase accuracy, all samples were analysed twice in two independent experiments to assess the interassay variations and to ensure reproducibility of the observed results (P > 0.05). No significant cross-reactivity or interference was observed.

### Statistical analysis

The results were analyzed by commercially available software package (Statview, Abacus concepts, inc., Berkley, CA, USA). The data were non-parametric, thus they were presented as median and interquartile range (IQR), which are between the 25^th ^and 75^th ^percentiles. Mann-Whitney test was used for comparison between these data. Chi-square test was used for comparison between qualitative variables of the studied groups. Spearman's rho correlation coefficient "r" was used to determine the relationship between different variables. For all tests, a probability (P) of less than 0.05 was considered significant. Patients were considered to have elevated serum neurokinin A or anti-ribosomal P protein antibodies if their levels were above the highest cut-off values (107.4 pg/ml and 92 units/ml, respectively) which were the 95^th ^percentiles of serum neurokinin A and anti-ribosomal P levels, respectively of healthy controls as the distribution of the data was non-parametric.

## Results

### Serum Neurokin A levels in autistic children and their relation to the degree of the severity of autism

Autistic children had significantly higher serum neurokinin A levels than healthy controls, P < 0.001 (table 1). Increased serum neurokinin A levels were found in 57.1% (40/70) of autistic patients.

**Table 1:** Serum levels of neurokinin A in autistic children and their relation to the severity of autism.

	Neurokinin A (pg/ml) Median (IQR)	Z (P-value)
Healthy children (n = 48)	52.5 (31)	3.5
Patients with autism (n = 70)	130 (328)	(< 0.001)
		
Patients with mild to moderate autism (n = 34)	54.5 (78)	5
Patients with severe autism (n = 36)	329 (649)	(< 0.001)

**Figure 1:**
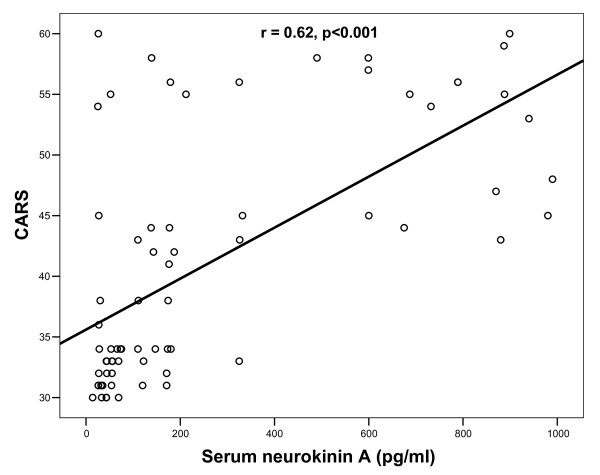
**Positive correlations between serum levels of neurokinin A and CARS in children with autism**. CARS: Childhood Autism Rating Scale.

Patients with severe autism had significantly higher serum neurokinin A levels than children with mild to moderate autism, P < 0.001 (table 1). Also, the frequency of increased serum neurokinin A levels was significantly higher in children with severe autism (31/36: 77.5%) than patients with mild to moderate autism (9/34: 26.5%), P < 0.001. Moreover, there were significant positive correlations between serum levels of neurokinin A and CARS in autistic patients, P < 0.001 (Figure 1).

Male and female autistic children had comparable values of serum neurokinin A (P = 0.52). In addition, serum neurokinin A levels had no significant correlations with the age of the children with autism (P = 0.68).

### The relationship between the elevated serum levels of neurokinin A and anti-ribosomal P protein antibodies in autistic children

**Figure 2:**
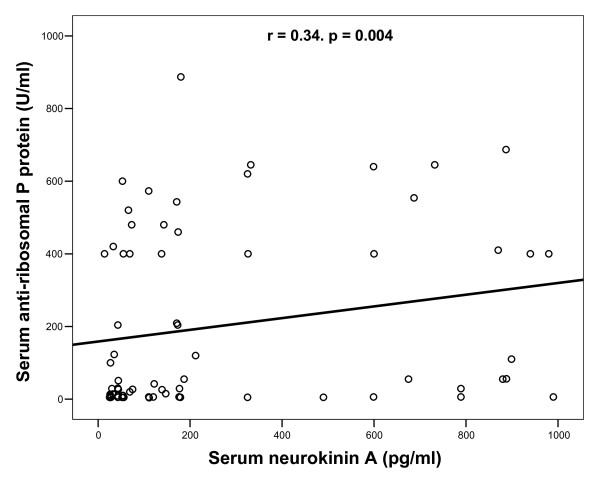
**Positive correlations between serum levels of neurokinin A and anti-ribosomal P protein antibodies in children with autism**.

Increased serum levels of anti-ribosomal P protein antibodies were found in 44.3% (31/70) of autistic patients. Patients with elevated serum neurokinin A levels had significantly higher serum levels of anti-ribosomal P protein antibodies [median (IQR): 115 (467) U/ml) than patients with normal serum neurokinin A levels [median (IQR): 23.5 (248) U/ml), P = 0.02. In addition, there were significant positive correlations between serum levels of neurokinin A and anti-ribosomal P protein antibodies in autistic patients, P = 0.004 (Figure 2).

## Discussion

Tachykinins are traditionally viewed as neuropeptides with well-defined functions as neurotransmitters. Tachykinin peptides have also an impact on the function of the immune system [32].

In our series, autistic children had significantly higher serum neurokinin A levels than healthy controls, P < 0.001. Increased serum neurokinin A levels were found in 57.1% of autistic patients. We could not trace data in the literature concerning neurokinin A levels in the blood of autistic patients to compare our results. This study was the first to investigate serum neurokinin A levels in autistic children.

In a recent study some neuropeptides were investigated in autistic children. It reported increased serum levels of neurotensin, while the other studied neuropeptides (β-endorphin and substance P) were not reported to be increased in these children [33]. Many studies reported that tachykinins may also be produced by non-neuronal cells, such as immune cells after exposure to inflammatory stimuli and they exert profound influence on the inflammatory responses by affecting multiple aspects of immune cell function [32]. In one study, the elevated sputum neurokinin A levels had significant positive correlations to eosinophil counts in both blood and sputum of asthmatic children during exacerbation [34]. Thus, the reason behind the increase of serum neurokinin A levels in autistic children may be the stimulation of the immune cells, after exposure to some environmental antigens (such as food allergens, infectious agents and heavy metals), with a subequent increase in the release of this tachykinin from these cells.

Neuroendocrine hormones, including tackykinins, triggered during stress may lead to immune dysregulation resulting from altered cytokine production, resulting in autoimmune or atopic diseases. Thus, the stress response with subsequent induction of a dysregulation of cytokine balance can trigger the hypothalamic-pituitary-adrenal axis and sympathetic nervous system through overproduction of neuropeptides and cytokines. In several autoimmune diseases such as rheumatoid arthritis, systemic lupus erythematosus (SLE), and diabetes mellitus, the immune dysregulation may be attributable to the neuroendocrine-immune network imbalance through overproduction of neuropeptides and cytokines [35]. One member of tachykinins, substance P, was reported to be increased in the cerebrospinal fluid obtained from patients with multiple sclerosis. Similar to autism, multiple sclerosis is an inflammatory disease of the CNS that is generally believed to represent an auto-immunological response to a component of myelin triggered by an environmental factor, in a genetically susceptible individual [36]. Substance P, through NK-1 receptors, contributes to the maintenance of CNS inflammation in multiple sclerosis. NK-1 antagonists, with the conventional anti-inflammatory treatments, may enhance the success of the treatment of some autoimmune diseases such as multiple sclerosis [37].

Tachykinin 1 gene is located in the candidate region for autism and produces substance P and neurokinins. These products modulate glutamatergic excitatory synaptic transmission and are also involved in inflammation which could be involved in the autistic brain. Therefore, tachykinin 1 gene may have some functions associated with the presumable pathophysiology of autism. To elucidate the genetic background of autism, one study analyzed the relationship between three single nucleotide polymorphisms of the tachykinin 1 gene and autism in the Japanese population, but no significant difference was observed between autistic children and healthy controls [38].

In the present work, patients with severe autism had significantly higher serum neurokinin A levels than children with mild to moderate autism, P < 0.001. Also, the frequency of increased serum neurokinin A levels was significantly higher in children with severe autism (77.5%) than patients with mild to moderate autism (26.5%), P < 0.001. Moreover, there were significant positive correlations between serum levels of neurokinin A and CARS in autistic patients, P < 0.001. This may indicate that the extent of the elevation of serum neurokinin A was closely linked to the degree of the severity of autism. However, it is not easy to determine whether the increase in serum neurokinin A levels is a mere consequence of autism or has a pathogenic role in the disease.

While glial cells are recognized for their roles in maintaining neuronal function, there is a growing evidence of the ability of resident glial cells to initiate and/or augment inflammation following exposure to allergens, trauma or infection in CNS. The tachykinins are found throughout the CNS, with an evidence for both neuronal and glial cells as being sources of them. Tachykinins are well known to augment inflammatory responses at peripheral sites, such as the gastrointestinal tract and skin, which raises the possibility that they might serve a similar function within the brain. Tachykinins may have a role in augmenting the immune functions of CNS glial cells resulting in the progression of damaging inflammation within the CNS [39]. Brain mast cells in some autoimmune neuroinflammatory diseases of CNS such as multiple sclerosis are activated by neural factors, including tachykinins. Mast cells can stimulate the activated T cells coming in contact with them at the blood-brain barrier In addition, brain mast cells secrete numerous proinflammatory and vasoactive molecules that can disrupt the blood-brain barrier, a finding that precedes clinical or pathologic signs of some autoimmune neuroinflammatory diseases of CNS [40].

Autoimmunity to CNS may have a pathogenic role in autism [41-44]. This may be indicated by the presence of brain-specific auto-antibodies in some autistic children [11-17]. In our series, increased serum levels of anti-ribosomal P protein antibodies were found in 44.3% of autistic patients. This study was the first to investigate serum levels of anti-ribosomal P protein antibodies in autistic children. Auto antibodies are the hallmark of autoimmune diseases. The reason behind the formation of some auto-antibodies in some patients with autism is not fully understood. It is speculated that autoimmune reaction might be trigged by cross-reacting antigens in the environment resulting in the release of some self antigens. These antigens may result in the induction of autoimmune reactions through the activation of inflammatory cells in genetically susceptible individuals [41,42].

Anti-ribosomal P protein antibodies are highly specific for SLE, especially for the neuropsychiatric manifestations including psychosis, mood disorders, anxiety, cognitive dysfunction and delirium [45]. A recent study has demonstrated a strong association between the seropositivity of anti-ribosomal P protein antibodies and the presence of neuropsychiatric manifestations in a group of children with SLE [46]. There are some studies in the literature relating anti-ribosomal P protein antibodies to the pathogenesis of organ damage in SLE. The main pathways described are cross-reaction with anti-dsDNA antibodies, a cytotoxic effect on mesangium cell proliferation, invasion into living cells and starting apoptosis, a defect in the synthesis of apolipoprotein B resulting in accumulation of lipids inside the cell, and downregulation of the total protein synthesis. P proteins are post-translationally modified (dephosphorylated) during apoptosis, and a dysregulation in the normal clearance of apoptotic cells leads to aberrant exposure of the immune system to modified non self-antigens. This could be one of the triggering events for the development of anti-P autoimmune response in some autoimmune diseases [45].

Moreover, in an experimental study, mice that had been received intra-cerebroventricular injection of anti-ribosomal P protein antibodies developed depression-like behaviors, which seems to be mediated by specific binding of these antibodies to limbic system brain areas, such as hippocampus and cingulate. It has been propsed that anti-ribosomal P protein antibodies both directly or indirectly affect CNS and produce a cytotoxic effect on neuronal cells. The mechanism by which these antibodies cross the blood brain barrier is unknown [47].

The potential role of neuropeptides in the progression and amplification of the immune neurogenic inflammation is of a great interest. These effects are described by the term immunological plasticity that include enhancement of survival, differentiation, and/or proliferation of immune cells and activation of the release of cytokines or mediators [48]. Therefore, neurogenic inflammation describes a vicious cycle of neuroimmune interactions that amplify immunogenic inflammation and neuropeptides are cross talks between the immune and nervous systems in immunogenic inflammation [49,50].

As neuropeptides were reported to have a possible role in some systemic autoimmune diseases and autoimmune neuroinflammatory diseases [35-37], we have tried to find a possible link between the elevated serum levels of neurokinin A and anti-ribosomal P protein antibodies in autism. In this work, patients with elevated serum neurokinin A levels had significantly higher serum levels of anti-ribosomal P protein antibodies than patients with normal serum neurokinin A levels, P = 0.02. In addition, there were significant positive correlations between serum levels of neurokinin A and anti-ribosomal P protein antibodies in autistic patients, P = 0.004. We could not trace data in the literature concerning the relationship between serum levels of neurokinin A and auto-antibodies in autistic patients to compare our results. We are the first to study such a relationship. The results of this study may indicate that the elevated serum levels of neurokinin A may be a possible contributing factor to the increased frequency of anti-ribosomal P protein antibodies in some autistic children. However, this is an initial report that warrants further research to determine the possible link between the elevated serum levels of neurokinin A and anti-ribosomal P protein antibodies.

After exposure to allergens, inflammatory cell (e.g. eosinophils) derived tachykinins are a major second source of these proinflammatory mediators [3,4] which can alter the function of the immune system [32]. Tachykinins may induce the so called neurogenic inflammation by recruitment and activation of the inflammatory cells [3,4]. Neuropeptides were reported to have modulatory effects on immune cells, in vivo, especially on T-helper (Th)1/Th2 balance. In addition, neuropeptides can directly stimulate lymphocytes to produce Th2 cytokines, going in line with the Th2 type shifted immune response [51-53]. Th2 cells orchestrate many aspects of pathologic immune responses including effector functions of B-cells, mast cells and eosinophils. These cells produce an array of cytokines such as IL4, IL-5, IL-9 and IL-13. In some autistic children there is an imbalance of T-helper (Th)1/Th2 subsets toward Th2, which are responsible for allergic response and production of antibodies [41].

Thus, the increased seum levels of neurokinin A may explain the increased frequency of anti-ribosomal P protein antibodies in some autistic children as a result of Th2 type shifted immune response. However, these data should be treated with a great caution, until further investigations are performed, as the seropositivity for anti-ribosomal P protein antibodies in some autistic children may be a mere association with the increased serum levels of neurokinin A in autism. A more detailed understanding of the interactions between tachykinins and immune cells may provide the basis for the development of new therapies for inflammatory and immune-mediated diseases [32]. Recent findings point to tachykinergic systems as promising targets of novel clinical agents in many inflammatory diseases [7]. These agents may antagonize NK2 receptors only [8] or may be NK1/2 receptor antagonists [9,10]

## Conclusions

Serum neurokinin A levels were elevated in some autistic children and they were significantly correlated to the severity of autism and to serum levels of anti-ribosomal P protein antibodies. However, this is an initial report that warrants further research to determine the pathogenic role of neurokinin A and its possible link to autoimmunity in autism. The therapeutic role of tachykinin receptor antagonists, a potential new class of anti-inflammatory medications, should also be studied in autism.

## Abbreviations

(CARS): Childhood Autism Rating Scale; (CNS): central nervous system; (IL): interleukin; (IQR): interquartile range; (Th): T-helper; (SLE): systemic lupus erythematosus.

## Competing interests

The authors declare that they have no competing interests.

## Authors' contributions

Both authors designed, performed and wrote the research. In addition, both authors read and approved the final manuscript.
